# The structure and genomic integration site of the HTLV-1 provirus determine selective clonal expansion and transformation to adult T cell leukaemia/lymphoma

**DOI:** 10.1186/1742-4690-11-S1-O76

**Published:** 2014-01-07

**Authors:** Lucy B Cook, Heather Niederer, Anat Melamed, Graham P Taylor, Masao Matsuoka, Charles RM Bangham

**Affiliations:** 1 Section of Immunology, Imperial College London, UK; 2 Section of Infectious Diseases, Imperial College London, UK; 3 Institute for Viral Research, Kyoto University, Japan

## 

Following infection and integration into the genome of host CD4+ T-lymphocytes, human T-lymphotropic virus type-1 (HTLV-1) persists by driving proliferation of infected T-cell clones which are counter-selected by HTLV-1-specific cytotoxic T-lymphocytes (CTLs). HTLV-1 Tax protein is the dominant target antigen recognized by these CTLs, and Tax is thought to cause the infected T-cell proliferation. In 40% of cases of adult T-cell leukaemia/lymphoma (ATLL) Tax protein is not expressed, suggesting that the provirus has down-regulated Tax to allow immune escape and viral persistence. Here, we tested the hypothesis that the genomic site of proviral integration determines the degree of clonal expansion and therefore determines the risk of ATLL. Proviral integration sites in 242 ATLL cases and 96 asymptomatic carriers were mapped and quantified using our recently described high-throughput technique (Gillet et al 2011, Blood, Cook et al 2012, Blood). In the ATLL cases, we identified promoter deletions predicted to cause tax gene silencing, we sequenced the tax gene and quantified promoter methylation. The results showed that 65% cases of ATLL could silence Tax by promoter deletion, tax gene nonsense mutations or tax promoter hypermethylation. High-throughput sequencing data showed that ATLL cases were not invariably monoclonal and that the provirus is typically integrated into a transcriptionally active region of the host genome. The results also suggest that transcriptional activity of the flanking host genome contributes to tax gene silencing, allowing CTL escape and clonal expansion.

